# The ethics of human-embryoids model: a call for consistency

**DOI:** 10.1007/s00109-021-02053-7

**Published:** 2021-04-01

**Authors:** Paola Nicolas, Fred Etoc, Ali H. Brivanlou

**Affiliations:** 1 Bioethics Center, New York Medical College, 40 Sunshine Cottage Rd, Valhalla, NY 10595 USA; 2 Laboratory of Stem Cell Biology and Molecular Embryology, The Rockefeller University, New York, NY 10065 USA

**Keywords:** Human-embryoids, Synthetic embryology, 14-day rule

## Abstract

In this article, we discuss the ethics of human embryoids, i.e., embryo-like structures made from pluripotent stem cells for modeling natural embryos. We argue that defining our social priorities is critical to design a consistent ethical guideline for research on those new entities. The absence of clear regulations on these emerging technologies stems from an unresolved debate surrounding natural human embryo research and one common opinion that one needs to solve the question of the moral status of the human embryo before regulating their surrogate. The recent NIH funding restrictions for research on human embryoids have made scientists even more unlikely to raise their voices. As a result, the scientific community has maintained a low profile while longing for a more favorable socio-political climate for their research. This article is a call for consistency among biomedical research on human materials, trying to position human embryoids within a spectrum of existing practice from stem cell research or IVF to research involving human subjects. We specifically note that the current practices in infertility clinics of freezing human embryos or disposing of them without any consideration for their potential benefits contradicts the assumption of special consideration for human material. Conversely, creating human embryoids for research purposes could ensure that no human material be used in vain, always serving humankind. We argue here that it is time to reconsider the full ban on embryo research (human embryos and embryoids) beyond the 14-day rule and that research on those entities should obey a sliding scale combining the completeness of the model (e.g., complete vs. partial) and the developmental stage: with more advanced completeness and developmental stage of the considered entity, being associated with more rigorous evaluation of societal benefits, statements of intention, and necessity of such research.

## Introduction

Scientific innovations often trigger fear. Over the last few years, research groups have reported striking progress on engineered models of human embryos derived from pluripotent stem cells [1]. As a result, media outlets have released sensational headlines detailing the manufacturing of synthetic humans in laboratories. The everyday reality of scientific research, however, is a far cry from those startling headlines. Moreover, the political climate in the USA has made it almost impossible to discuss human embryoids’ ethical status without immediately opening the door to a broader discussion about abortion. The fervor around this debate has contributed to a mischaracterization of these complex technological innovations. Despite efforts to engage in meaningful technical discussions, the debate always falls back to the vast and unsolvable debate around the moral sanctity of the human embryo.

The lack of a federal policy on human embryonic research is largely due to the lack of consensus regarding the moral status of the embryo [2]. Despite the absence of federal policy, the “14-day rule,” which limits research on human embryos beyond 14 days, has been followed since 1979 and is nearly universally accepted in reproductive medicine [3]. Restrictions on NIH funding for research involving human embryos, human fetal tissues, and human embryoids has made scientists unlikely to raise their voices, maintaining a low profile while longing for a more favorable socio-political climate.

This article is a call for consistency, positioning human embryoids within a spectrum of existing practice from stem cell research or IVF to research involving human subjects. We distinguish between ethical concerns that are raised by different kinds of human embryoids that are (1) partially mimicking embryonic parts or (2) the integrated development of the embryo as a whole. It is clear that the 14-day rule cannot apply to the first category (partial embryo model) as these models clearly lack the potential to form a full organism, but that discussions are needed to determine the ethical framework of (near-)complete embryo models, especially when these models have the potential to implant in utero (e.g., blastoids). Currently, complete models of the human embryo have not been generated. However, in order to anticipate on such ongoing research, here we propose arguments and parameters so as to address the most pressing questions relative to (1) whether human blastoids should be transferred into an uterus and (2) how long should we culture in a dish human models that are complete (e.g., blastoid) or incomplete but that form a complete epiblast (e.g., ETX). We argue that it is time to reconsider the full ban on embryonic research (intact embryos and embryo-like structures) beyond the 14-day rule and that research on those entities should obey a sliding scale: the more advanced the stage of the intact embryos or embryo-like structures, the more scientific justifications, societal benefits, and oversight are needed.

## Current status of human embryoids research

The derivation of human and mouse embryonic stem cells has provided the opportunity to work with embryonic building blocks. One exciting challenge is the attempt to discover the minimal set of physical and chemical conditions that can trigger their self-organization in embryoids. Embryoids are multicellular assemblies resembling natural embryos, not only in terms of cell types but also in 3D organization. This embryological “bottom-up” approach that proposes to re-build embryos from their basic components has the potential to reveal the fundamental principles governing mammalian embryogenesis, with implications in reproductive and regenerative medicine. In this article, we use the term “embryoids” to refer to all structures made from stem cells aggregates to reconstitute a large part of the human embryo comprising of multiple embryonic cell types, regardless of developmental stage.

**Fig. 1:**
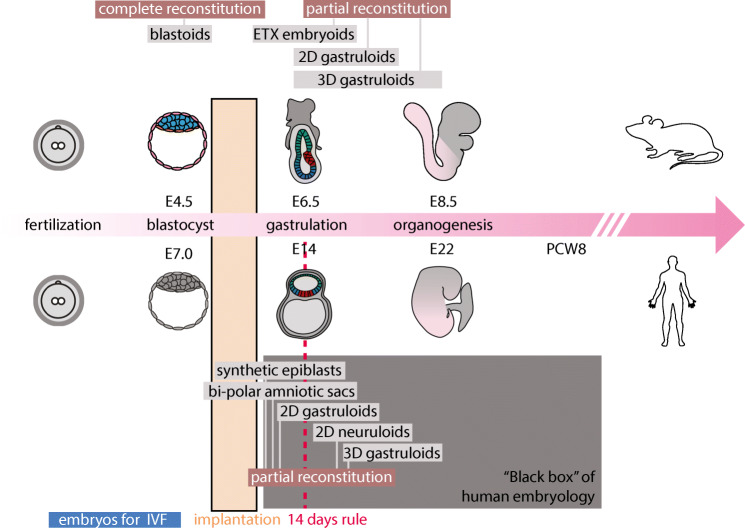
Timeline of human and mouse early development. Embryoids that are partial reconstitution or complete reconstitution of human and mouse embryos are placed along the developmental timeline. Only colored tissues have been reconstituted in embryoids while the gray tissues are still lacking from these models. Moreover, embryos discarded from IVF procedures, as well as the time of implantation and the 14-day rule, are indicated. Adapted from [4]

Despite vast temporal and morphological differences, mouse and human development follows similar stages (Fig. 1). Very briefly, following fertilization, the single-celled egg rapidly divides into a ball of cells that condenses and quickly segregate into three cell types that form the *blastocyst*. At the pre-implantation blastocyst stage, the two extra-embryonic lineages and the epiblast lineage are formed and co-develop by exchanging molecular signals [5, 6]. The two extra-embryonic tissues of the blastocyst, which are termed trophectoderm and primitive endoderm, are the precursors of many transient developmental tissues that ultimately form the placenta and the yolk sac, respectively. Concomitantly, the epiblast lineage is formed, which is the pluripotent precursor of the whole fetus that ultimately forms the whole body. The fertilized mammalian egg thus produces not only the fetus but also extra-embryonic tissues/organs that are essential to (1) mediate the attachment and implantation into the uterus (trophectoderm of the blastocyst), and (2) support the further development of the fetus (e.g., placenta). Without extra embryonic tissues, the blastocyst cannot implant nor develop in utero. Once the embryonic and extra-embryonic lineages have formed and the blastocyst has implanted, these extra-embryonic tissues co-develop in synergy with the epiblast, and guide it to enter a process termed *gastrulation*. Note that it has been shown lately that the primitive endoderm also contributes to the formation of specific foetal tissues [7]. Each cell type rapidly expands, and implantation into the uterine wall takes place. During gastrulation a transient structure called *the primitive streak* is formed that breaks the radial symmetry of the embryonic disk (*epiblast*) and leads to the emergence of the three embryonic germ layer derivatives: *ectoderm*, *mesoderm*, and *endoderm* laying out the foundations of the body plan and the definition of the embryonic axes. The descendants of the embryonic germ layer will generate all the organs of the adult. For example, ectoderm gives rise to the entire nervous system, sensory organs, and skin; mesoderm to blood, heart, muscle, and bone; and endoderm to lung, gut, pancreas, and liver. Subsequent morphogenesis within the three germ layers will provide the foundation for the different organs that will continue their growth to eventually become mature and functional (Fig. 1).

From this short description, it is evident that rebuilding an embryo from scratch is a daunting task. First, there is an obvious architectural challenge as embryos at any stage are comprised of multiple cell types which are arranged in a peculiar architecture. Second, and perhaps more challenging, early embryonic development is highly dynamic. The shifts in shape that occur during the first weeks of development are the most dramatic changes that will occur in an organism’s lifetime. Despite these impediments, we project that in the future, it will be technically feasible to grow embryoids, built through defined bioengineering methods, that will be able to continue on to later development stages.

Within this framework, synthetic embryology has become a vivid research area in the last decade, and we refer the reader to a recent review on the subject [1]. Since the mouse has historically been the model of choice for mammalian embryology, it is without surprise that mouse embryoids are currently significantly more advanced than human ones. We can distinguish between two types of embryoids: the ones aiming at a complete reconstitution of a specific embryonic stage, with all cell types present in the correct geometry; and the ones presenting a partial reconstitution of an embryo with only a subset of the cell types present at a particular embryonic stage. The concept of regenerating a nearly complete embryo from pluripotent embryonic cells has its roots in classical experimental embryology studies of the amphibian system which ultimately lead to the cloning of the first animal: a frog [8]. These self-organizing embryo models which can be obtained from different throughputs are precious tools for modern human embryologists as they provide an unlimited source of biological material to explore periods of our development that would otherwise be impossible to scrutinize.

In mammals, the epiblast cannot develop without the support of the two extra-embryonic tissues (trophectoderm and primitive endoderm). To date, only few studies have reported the creation of embryo models including these extra-embryonic tissues or their descendants. At the moment, such a complete model has only be generated at the blastocyst stage [5, 9, 10] (blastoid), and crucially, with mouse stem cells, not with human stem cells (see Fig. 1; the tissue that have been modelled are colored, while the full conceptus is in gray). This leverage the possibility to create, from mouse embryonic stem cells, independent and stable cultures of the different embryonic and extra-embryonic cell types which constitute the blastocyst. This has facilitated the creation of embryoids through a mixing approach where the different cell types are prepared independently and mixed under specific conditions. Remarkably, once the different embryonic and extra-embryonic cell types are brought together they are able to self-assemble into embryonic geometries with varying efficiency. As of yet, blastoids represent the only successful attempts at the creating complete mouse embryoids that could be implanted to initiate a pregnancy in mice. Another model, termed ETX, forms analogs of some inner post-implantation tissues, namely the epiblast, visceral endoderm, and extra-embryonic ectoderm, but misses key outer extra-embryonic tissues such as the parietal endoderm, parietal trophectoderm, and ectoplacental cone. The precursors of these outer extra-embryonic tissues (the trophectoderm and primitive endoderm) mediate attachment and implantation of the blastocyst into the uterus and support its in utero development. As such, only blastoids recapitulate the pre-implantation stage and form the extra-embryonic tissues that are necessary to recapitulate aspects of implantation upon in utero transfer. Of note, this interaction with the uterus is essential to support the progression through the subsequent stages of development and thus, despite some potential for autonomous development [11] the uterine environment endows the blastocyst with the potential to form an organism. Still, these structures could not reach later developmental steps in vivo for unknown reasons. It should also be noted that all current mouse or human embryoids become disorganized after a few days in culture or in vivo, and only model a short window of development. Therefore, it is important to realize that we are currently very far away from embryoids that can form a full organism, both in the mouse and human case, demonstrating an absence of organismal potential of the current state of the art.

Three popular methods have achieved a “partial reconstitution” of the human or mouse embryo starting at gastrulation stages:

- 2D gastruloids leverage micropatterning techniques to constrain pluripotent stem cells into colonies of defined shapes and sizes [12]. In this setting, geometric confinement is sufficient to create dynamic signaling waves that induce the radial self-organization of the germ layers [13]. While extra-embryonic fates are found in this system, the geometry is 2D, and it lacks the reconstitution of the gastrula’s architecture. An extension of this method has produced neuruloids that are a very accurate recapitulation of the ectodermal compartment, post gastrulation at neurulation stages, complete with the four main ectodermal derivatives: neural tissue, neural crest, sensory placode, and skin juxtaposed in similar pattern distributions as in the natural embryo [14].
- The second type of embryoid focuses solely on the core of the embryo: the epiblast. This approach models synthetic epiblast as a sphere, which can be induced for gastrulation by applying morphogens [15, 16]. This model is only a partial representation of an embryo as neural tissues are completely missing as well as all extra-embryonic lineages.
- Finally, 3D gastruloids rely on pluripotent stem cell aggregates induced for differentiation by a pulse of growth factor and have been derived from both mouse [17] and human [18] cells. 3D gastruloids offer an axial organization reminiscent of the anterior-posterior embryonic axis. They provide impressive models of mesodermal development with cell movements leading to the elongation of the embryonic axis and formation of muscles (somitogenesis [17]). Like synthetic epiblasts, they are a partial representation of an embryo as they lack neural tissues and extra-embryonic derivatives.

While some headlines report the creation of viable embryoids that can be turned into human beings, the reality behind the published methods are, for the moment at least, less dramatic. First, most of the work in the field focuses on the partial reconstitution of embryonic parts. These models are therefore unable to turn into a human being. As mentioned above, completely reconstituted embryoids have been reported only in the mouse, and only at the blastocyst stage, preimplantation. Moreover, while these embryoids were able to develop a little further and initiate implantation upon placement into the mouse uterus, they stop developing very quickly and are unable to become viable offspring: blastoids are currently incapable of reaching the gastrula stage and ETX models are incomplete as they do not form multiple outer extra-embryonic tissues, especially the ones that make the connection with the mother. Without any doubt, it only a matter of time until the mouse protocols are adapted for the formation of human blastoids. To adapt protocols developed for the complete reconstitution of embryos in the mouse to the human, methods to obtain human extra-embryonic cells are required, and are being developed at a quick pace [19, 20]. Once human blastoids are created, it would be theoretically possible to transfer them into a uterus, as for them to implant and further develop. However, the road to successful development would, most likely, be very long. Just as with stem-cell based regenerative therapies: as complex projects such as neuronal transplantation therapies start to get closer to the clinics [21], we realize that they had to overcome immense technical hurdles to reach satisfactory clinical standards. We thus envision that the potential prospect of transferring human blastoids into a uterus with the intend of inducing a pregnancy would have to follow a similarly long and difficult timeline before being successful. In conclusion, building a preimplantation human embryoid which would be theoretically ready for implantation in a human uterus is within reach, but making such an embryoid competent for a successful pregnancy would take a long and extensive period of clinical research and would require setting up complex clinical trials which would be very likely to lead to abnormal fetuses. Consequently, from both technical and ethical perspective creating offspring from embryoid is off the table.

## The current state of regulation on human embryoids

Currently, there are few explicit regulations regarding human embryoids [22]. Different jurisdictions do not necessarily share the same definition of an embryo, and often do not include an explicit definition, leaving us with uncertainty regarding the how to view human embryo models [4]. Not only is it difficult to apply the notion of human organismal potential to embryo models, but it is also unclear to what extent the 14-day rule applies to human embryoids. In particular, it is unclear how the 14-day rule translates to *in vitro* embryoids which are known to have different timing and can bypass the developmental timeline of normal embryos. For instance, human pluripotent stem cells grown in micropatterns can form structures resembling the primitive streak stage within 48 h of culture and have a starting point made from pluripotent stem cells of epiblast nature which can only be loosely timed to an embryo in a window between day 7 and day 12. Since reaching a conclusion on the timing of these structures is not straightforward, the simple translation of the 14-day rule to these new entities is not advisable.

The 2016 guideline of the International Society for Stem Cell Research (ISSCR; Box 1) suggests that research involving embryoids that might manifest “human organismal potential” be reviewed by a proposed human Embryo Research Oversight (EMRO) process and prohibited if they violate the 14-day rule” [23].

**Table:** Box 1. Sections of current International Society for Stem Cell Research guidelines relevant to human embryoids [23]

Forms of research that are permissible only after review by an EMRO process Research involving the *in vitro* culture of embryos or experimental generation of embryo-like structures that might manifest human organismal potential, to ensure minimal periods of *in vitro* culture, as justified by compelling scientific rationale. Prohibited research activities 1. In vitro culture of any intact human pre-implantation embryo or organized embryo-like cellular structure with human organismal potential, regardless of derivation method, beyond 14 days or formation of the primitive streak, whichever occurs first. 2. Experiments whereby human embryos or organized cellular structures that might manifest human organismal potential are gestated ex utero or in any non-human animal uterus.	

Moreover, it is unclear how the Dickey-Wicker Amendment banning federal funding for “the creation of a human embryo or embryos for research purposes” or “research in which a human embryo or embryos are destroyed, discarded” [24] applies to embryoids. The amendment impacts all agencies within the Department of Health and Human Services, including the National Institutes of Health (NIH). The NIH’s explicit policy does not state that it won’t support research to create or use models of human embryos, sometimes referred to as “human embryoids” or “human gastruloids,” rather: “if the cells could be considered an organism, as described in the NIH Grants Policy Statement Section 4.2.5, then such research is subject to the limitations described in Section 4.2.5. These considerations are made on a case-by-case basis (see Box 2).” However, because of the absence of consensus regarding applying existing regulations to those new entities, the NIH recently refused to fund research on embryonic models and embryo-like structures such as 2D gastruloids.

**Table:** Box 2. Sections of current NIH policies for research on embryoids

**4.2.5 Human Embryo Research and Cloning Ban** NIH funds may not be used for (1) the creation of a human embryo or embryos for research purposes; or (2) for research in which a human embryo or embryos are destroyed, discarded, or knowingly subjected to risk of injury or death greater than that allowed for research on fetuses in utero under 45 CFR 46.204(b) and subsection 498(b) of the PHS Act (42 U.S.C. 289g(b)). The term “human embryo or embryos” includes any organism not protected as a human subject under 45 CFR 46, as of the date of enactment of the governing appropriations act, that is derived by fertilization, parthenogenesis, cloning, or any other means from one or more human gametes or human diploid cells. Furthermore, per the NIH Director's Statement of April 28, 2015, NIH will not fund any use of gene-editing technologies in human embryos. In addition to the statutory restrictions on human fetal research under subsection 498((b) of the PHS Act, by Presidential memorandum of March 4, 1997, NIH is prohibited from using Federal funds for cloning of human beings.	

## Reframing the debate: two dead-ends

Should the human embryoids share the same ethical status and be regulated the same way as intact human embryos? Such framing assumes that we need to know (1) the moral status of a natural embryo and (2) whether human embryoids are more than a model and should fall into the same category as natural embryos, i.e. do they have organismal potential?

### The absence of consensus regarding the moral status of the embryo

The debate over the moral status of a natural embryo has always been a minefield and encompasses a vast spectrum of positions [25]. Some consider that moral status is binary: an entity has full moral status with a set of rights, or it does not [26]. Others view moral status as developmentally emergent [27, 28]. From the view that they have full moral status starting at conception, to the view that embryos acquire more moral status through their development or that they have no moral status whatsoever, no consensus can be found among community members or religious groups. Disagreements about the moral status of an embryo exist even within the strict boundaries of the Roman Church orthodoxy [29]. Despite the current consensus within the Catholic Church that human life starts at conception, some theologians actually posit that the moral status is biologically emergent. According to Aquinas, it is only once the fetus has become animated by the rational soul that it is homicide to kill it [30].

The debate around the moral status of the embryo and the fetus relies so deeply on individual and societal values that the prospects for reaching a consensus between stark and entrenched oppositions look quite dim [31]. In a striking demonstration of the absence of consensus, none of the established guidelines cited above, from the 14-day rule itself to the ISSCR recommendations, are based on moral status. The Warnock Committee explicitly refused to address the question of moral status or whether an embryo should be considered a person [32]. In selecting 14 days as a limit, the Warnock Committee referred to sentience (defined at minima by the ability to experience pleasure and pain) and individuation as relevant factors [32–34]. By doing so, the committee acknowledged the existence of morally signifying traits, and the necessity to place some limits on human embryonic research while being realistic regarding the possibility of knowing with certainty when the cutoff must be set. The 14-day rule is the product of a reasonable accommodation between dissenting parties with opposite views [35]. It has the benefit of providing a rather clear and actionable developmental limit for the culture of the natural embryo, without intending to rely on moral values. Why, then, would we contest this pragmatic compromise that has such benefits [32, 36, 37]? Recent research developments mandate the reevaluation of the balance between accommodating diverse moral views in our society and supporting scientific innovations that could potentially bring therapeutic benefits. As stated by Chan: “Policies made in controversial areas should be open to change, recognizing the possibility of evolving norms and moral thinking” [32] and progress made from the drafting of the 14 day-rule in 1978 needs to be revised to build an acceptable compromise taking into account the updates of scientific innovation [38].

### The absence of consensus regarding the model problem

Another controversial question is whether embryoids are more than a model and, consequently, share the same ethical status as an intact natural embryo. One can start answering this question by making extensive side-by-side comparison between embryoids and natural embryos. This can be achieved at a quantitative level in a number of ways, from examining the molecular signature of the different cell type, to the comparing the shape of the tissues and global morphologies. However, as these in vitro structures become more and more complex and their analytical description more and more detailed, the harder it becomes to draw a line and know when similarity becomes an identity of nature. Scholars have emphasized the vicious circularity of the model question: human embryo models are designed to avoid experiments on human embryos, but more research on human embryos is needed to know whether embryoids are just a paradigm or more than a model [4, 39]. We now need to have an in vivo equivalent to our embryoids to assess the degree of accuracy and what stage of development the models correspond. In the absence of these controls, and assuming that we would be successful in growing these embryos past day 14, it would be very difficult to determine how well the models faithfully represent natural embryological events that occur during that stage. The definitive functional proof—the implantation and development of a human embryoid in a natural maternal womb—is out of the question. Even if this was technically feasible, many questions remain on whether embryoids that are likely to generate abnormal organisms should be ever transferred in utero and how such techniques would eventually be assessed through clinical trials.

First, generally speaking, we argue that the potential of an embryo, and by extension of an embryo model, does not reside in its composition (e.g., cellular and molecular), but rather emerges when it is combined with a supporting extrinsic environment. In other words, because the extrinsic environment (e.g., an uterus or an engineered bioreactor) is absolutely required to support development, we argue that embryos and embryo models only become endowed with a potentiality when combined with an extrinsic environment that support their development until a certain stage.

The “model problem” (are embryoids more than a model?) is intrinsically related to the way we conceive of a “human organismal potential” and the way we interpret “potentiality”, that is, do embryoids have the potential to develop into a full human being? Arguments that rely on the notion of potentiality claim that if “entity *x* has the potential to develop into *y*”, then the entity *x* has some degree of moral status [40], compared to an entity that would have no potential to develop into *y* [41]. The discussion around human organismal potential has been particularly contentious. First, some have argued that it is not because *x* may potentially develop into *y* that *x* currently has the “moral rights” of an actual *y*. Furthermore, the case of somatic cells shows that not all forms of potentiality are morally significant [42, 43]. As stated in Baertschi et al., “the more it becomes obvious that somatic cells have the capability to be restored to embryonic stem cell state and the more indistinguishable embryonic cells become from somatic cells in terms of potentiality, the harder it becomes to see what is so special, in ethical terms, about embryonic cells and embryos” [44]. Consequently, scholars have argued that the notion of human organismal potential is too broad to help regulate those new entities meaningfully. Scholars have proposed [32, 45], for instance, to refine the notion of potentiality by distinguishing an active potential where an entity’s development is only determined by internal factors, by opposition to a “passive potential” requiring at least one extrinsic condition to fully develop [41]. Strictly speaking, only embryos in utero would have an active potential to develop into a fully mature human being [45]. In the category of passive potentiality, a distinction should be made between entities that miss an essential part of embryonic development (synthetic epiblast are only a partial representation of an embryo as neural tissues are completely missing as well as all extra-embryonic lineages) and entities that also comprise both of embryonic germ cells lines and extra-embryonic tissues. We agree with Pereira Daoud et al. that “this need not mean that there are no grounds for ascribing moral status to pre-implantation embryos. Doing so (…) must then be a matter of granting early human life a certain degree of symbolic value or moral standing by association, rather than acknowledging any intrinsic moral value” [46].

The scientific design intends to distinguish different types of potentialities in the theoretical hypothesis that such embryoids would perfectly mimic the development of a part of the embryo or the embryo as a whole. As mentioned above, scholars recently called to “include the degrees of potential of human embryoids for moral consideration” “as many countries have based their regulation of research using human embryos on the potential to develop into a human being” [41]. As previously proposed, researchers make a distinction between “constructs that do not attempt to model the integrated development” of the embryo as a whole (e.g., gastruloids, epiblast-amniotic sac structures, or micro-patterned stem cell cultures) and “experimental constructs that attempt to model the integrated development” of the embryo (e.g., blastoids, ETX embryoids) [4]. Those distinctions should inform new NIH regulations: the notion of human organismal potential, as currently used by NIH guideline without further specifications, lacks the sophistication necessary for regulating different types of embryoids.

## Call for consistency

There are currently more than 1 million cryopreserved embryos in IVF centers and cryobanks. Ninety thousand are likely abandoned every year [47, 48]. The sheer number of cryopreserved embryos means that it is only a matter of time until perpetual maintenance of abandoned embryos becomes unaffordable for cryobanks. “Those embryos currently are either treated as mere waste or maintained cryopreserved until their inevitable destruction” and, thus, are never used for research purposes but rather left “abandoned” [48]. Scholars have consequently proposed establishing non-profit human embryo banks for research use since “a higher purpose, surrogate consent and appropriate public oversight would indeed represent a far more respectful use of abandoned human embryos.” In a similar vein, philosophers such as Sissela Bok [49], Thomas Douglas, Julian Savulescu [50], and Michael Sandel [51] have argued that there is so much embryo loss occurring naturally during the first stages of pregnancy [52] that if we were seriously concerned about embryo loss, “it would be necessary to undertake a monumental struggle against all miscarriages.” A staggering 40% of human embryos fail to implant into the uterus. We, therefore, arrive at a paradoxical situation where we have been concerned with the ethicality of embryoids while our society does not consider the large numbers of discarded frozen embryos from IVF or embryo loss from early miscarriages to be on the same level. However, research using embryoids has the potential to find treatments for infertility, reducing the number of embryos in successful IVF cycles, reducing overall abandoned embryo numbers, and avoiding miscarriages. In other words, creating in vitro models solely for research purposes is not ethically more problematic than letting frozen embryos accumulate without any utility or benefits for our society. The current practice of freezing human embryos or disposing of them without considering their potential benefits actually contradicts the assumption of special consideration for human material. Conversely, creating embryoids for research purposes acknowledges that no human material should be used in vain nor without scrutiny but should always serve humankind in important researches and represent both a powerful technical and ethical alternative to the use of natural embryos for research.

Federal regulations do not consider ex vivo embryos “human subjects” (section 1, see 45 CFR 46). However, let’s assume for our argument that a 14-day embryo already has full moral status and therefore deserves the same protection as a person. This status does not necessarily prohibit any experimentation or research on the embryo. For instance, research on human subjects shows that the moral dignity of a person does not in itself preclude any research or experimentation. It necessitates, however, a stringent and coherent framework for evaluating the ethics of research studies. Our society has defined informed consent, along with other principles, such as transparency, proportionate risks, and potential benefits, as a requirement for experimentation on human subjects. We call for moral consistency in the way our society treats human material: research on human embryoids is not necessarily more morally problematic than research involving human subjects (regulated by a set of requirements) or existing practices such as freezing human embryos without any potential benefits for our society.

### Defining a consistent social project

As previously stated, it is unrealistic to expect consensus on the moral status of the embryo (natural or embryoid), as this question cannot be answered without a philosophical framework that goes well beyond the strict boundaries of biology. Since parties do not share the same premises for their argumentation, a public debate over the embryo’s moral status is unlikely to help legislators craft future regulation. This does not mean that the question of the moral status of the human embryo is socially irrelevant. Each party holds positions that contribute to maintaining an equilibrium in the way we frame the issue. These dynamic tensions remind us that we must show the utmost caution and vigilance when it comes to study human material. The question of “how my beliefs and values inform where I think we should draw the line for experimenting on human embryos” is quite different than the question of “what should we do as a society in regard to them?” Because we live in a pluralistic society, we need to define our social priorities and decide on new rules that can keep pace with recent scientific developments while leaving room for therapeutic and clinical advancements that will benefit our society as a whole. Still, it is crucial that we acknowledge that limitations must be drawn [53]. Accordingly, we also argue that we should revise the current legislation for federal funding on human embryo research.

### 1. Partial embryonic reconstitutions.

The present state of the art protocols only provide partial and incomplete reconstitution of the intact human embryo. This encompasses research that does not include all cell lineages (e.g., synthetic epiblast, 3D gastruloids, amniotic sac structures) or models that lack essential traits, like the 3D architecture of the embryo (e.g., 2D gastruloids). Consequently, they are not equivalent to intact embryos. Strictly speaking, they don’t have any organismal potential. Those culture systems do not attempt to model a complete embryo-like structure but to study a window of development that does not comprise all essential internal factors to be considered autonomously self-organizing [4]. As such, the 14-day rule should not be applied to those entities.

Other ethical issues may arise if those incomplete entities that do not necessarily comprise all canonical steps of embryogenesis attempt to model functional organs that could develop morally signifying traits. The case of cerebral organoids has drawn public attention in regards to the apparition of sentience or consciousness. This important discussion is out of the scope of this paper as we only focus on embryoids that mimic the first stages of embryological development [39].

### 2. Ethical considerations about complete embryonic reconstitution before and after the “14-day rule”

The current 14-day rule, if applied to embryoids, allows for models that attempt to mimic human development up to the stage of the primitive streak. However, there remains a need to address future ethical concerns raised by complete embryoids that could eventually model human embryonic development after the formation of the primitive streak. If implantation in the uterus is not to be considered, these developments will most likely require an artificial membrane to mimic implantation in the uterine wall.

The 2016 ISSCR guidelines based the prohibition of research on embryoids after 14 days on a “broad international consensus that such experiments lack a compelling scientific rationale, raise substantial ethical concerns and/or are illegal in many jurisdictions.” We argue that we should re-open this debate as: (1) the 14-day rule is arbitrary. In section A, we argued that consensus is not so much around the content of such rule than it is about acknowledging that we benefit from having a rule, even if it is arbitrary. One may wonder, “What are we afraid of, in contemplating the possibility of crossing this limit? (…) Certainly, the post-14-day embryo does not suddenly become something inviolable or deserving of moral protection” [32]. Consequently, we should propose a new arbitrary rule that would have greater benefits for scientific innovations. (2) We now see the potential medical benefits of studying both embryos and embryo-like structures after 14 days. Besides obvious applications to fertility issues, the origins of developmental disorders remain largely unknown, and technicalities solved here will most likely pave the way for organ creation for regenerative therapies. Early congenital diseases, or even late onset diseases have their roots in early embryogenesis. Autism is a case in point, understanding the cause of the disease requires fundamental knowledge about what happens during the earliest stages of neural development and neural functions. However, the earliest human tissues available for research are aborted human fetuses, which are most of the time retrieved around post-conception week 11-13 at the earliest. One can think of this period between 11–13 weeks and 14 days as a black box that contains crucial knowledge about the pathophysiology of developmental disorders for which embryoids could provide invaluable information. In the first step, we propose extending research both on human embryoids and natural human embryos up to 28 days, as the sensory system is too immature at this stage for sentience or pain to be established [36, 38]. This shift could be the beginning of conceptualizing a sliding-scale framework that will weight research benefits versus risks on a case-by-case basis.

We conclude that we should discuss the possibility of creating human embryoids that attempt to model the integrated development of an embryo after 14 days. As previously stated, there is are no current reports of complete human embryoids that could present implantation potential. Further, no embryoid has been endowed with organismal potential. As such, embryoid research should be allowed not only before but after 14 days. An extended set of rules must be written that would both serve humankind and be aligned with the acknowledgment that no human material (intact embryos or embryo-like structures) should be used without scrutiny and oversight. We further argue that as a society, we should revise the ban of federal funding for the creation of complete human embryoids or the use of embryos for research purposes.

## Recommendations for future guidelines for research on embryo-like structures after 14 days

Research on complete embryoids after 14 days should be strictly regulated and follow a set of ethical principles. Research should obey a sliding scale: the more advanced the stage of the embryo-like structures, the more scientific justifications, societal benefits and oversight, evaluation of the value, the intention, the necessity, the risks and the benefits of the research project, are needed. Here, we are adapting the 2016 ISSCR to those new entities and using scholars’ previous recommendations [23].

1. Value—enhancements of health or knowledge must be derived from the research. To be ethical, research on embryo-like structures must be valuable, meaning that the scientific project must test a hypothesis that can generate important knowledge about human development. It does not entail that research on those entities must have immediate practical ramifications. Fundamental research is essential to medical and clinical innovations. It means that the value of research depends intrinsically on a definition of our social and medical priorities and that research must move forward accordingly to priorities defined by our community.
2. Intention of the project—“*The intention of the research* should be considered the key ethical criterion by regulators” [54]. We agree that, as previously proposed [4], “researchers can help avert the potential for moral confusion by incorporating elements of engineering ethics early in their work.” What are the commitments taken by the scientific community to place boundaries to their own work? As part of this contract, human embryoid models should not be created for direct use in assisted reproduction aimed at producing a pregnancy. They could be used as in vitro models for better understanding infertility issues. Studying a discrete and defined period of development or a discrete set of anatomic structures, rather than modeling the continuous development of an intact embryo or fetus should not fall into the same category. They do not raise the same ethical concerns.
3. Necessity—The research project should avoid, when it is possible, using means that are unnecessary. Project proposals should include a discussion of alternative methods. If it is possible to study a *discrete* and *defined* period of development of a discrete set of anatomic structures rather than modeling the continuous development of an intact embryo or fetus, this approach should be preferred. For research questions that cannot be fully answered without modeling a *continuous integrated* development after 14 days, culture systems that model the integrated development of the embryo after gastrulation should require a mandatory review.
4. Risks/benefits—For culture systems that could model the integrated development of the embryo after the apparition of the primitive streak, we need to open a public debate to decide (a) how far are we willing to go *depending* on the potential benefits for clinical and medical research and (b) how to mitigate the potential benefits with the potential risks. We discussed the absence of consensus and certainty regarding the moral status of the embryo and the stages at which it presents signifying moral features, but we must at least acknowledge that the more advanced the developmental stage of embryo-like structures and their completeness, the more likely they are to exhibit some signifying moral features. In other words, the risk that an embryoid at a later stage of development may exhibit some signifying moral features must be part of the conversation. The presence of such moral features does not necessarily prohibit any kind of research. Such level of risk (different at each stage of development) must be mitigated depending on the clarity and the evidence of future potential benefit.
5. Transparency and oversight—The comprehensive ISSCR guidelines show the importance of rigorous monitoring by a specialized human embryo research oversight (EMRO) process. Oversight must be provided depending on the kind of embryo-like entities (complete/partial; before or after the 14 days).

## Conclusions

This article calls for shifting the debate surrounding embryoids towards pragmatism. Although, at the present state of the art, none of these models has yet been demonstrated to develop for more than a few days in vitro, we can envision that cell culture methodologies could be refined to a point where the models capture key features of early human development and minimize differences with the natural embryo. We insist on making a clear distinction between our individual values and our societal values. We need to keep up with the pace of science and design an ethical framework that will regulate these entities despite the absence of clear consensus about the moral status of the intact embryo. The recommendations suggested above are only the beginning of an open public debate between scientists, research policy experts, bioethicists, and community members for extending the 14-day rule and revising the Dickey-Wicker Amendment.
